# Marked improvement of neuropsychiatric symptoms following control of allergy symptoms with the use of humanized murine anti-IgE antibody (omalizumab) in 2 patients with severely limited expressive language

**DOI:** 10.1186/s13223-015-0105-x

**Published:** 2015-12-09

**Authors:** Harumi Jyonouchi

**Affiliations:** Pediatric Allergy/Immunology, Department of Pediatrics, Children’s Hospital at Saint Peter’s University Hospital, 254 Easton Ave., New Brunswick, NJ 08901 USA

**Keywords:** Allergic rhinitis, Neuropsychiatric symptoms, Limited expressive language (LEL)

## Abstract

Flare-up of allergic rhinitis has been implicated in worsening neuropsychiatric symptoms such as hyperactivity and anxiety in the general population, mostly supported by epidemiological data. However, it is unknown how such respiratory allergy symptoms affect behavioral symptoms in patients with intellectual disability and limited expressive language. These patients may express more severe behavioral symptoms partly due to frustration and anxiety, being under-diagnosed and undertreated secondary to a lack of proper communication means. Herein, we present two cases of patients with severely limited expressive language , in whom we observed marked improvement in behavioral symptoms and even cognitive activity following control of their symptoms of allergic rhinitis with the use of omalizmab, a humanized anti-IgE antibody. The presented cases indicate that clinicians need to be aware of profound effects of allergy rhinitis on neuropsychiatric symptoms in individuals with limited expressive language.

## Background

The prevalence of immunoglobulin E (IgE)-mediated allergic diseases such as allergic rhinitis (AR) and atopic asthma is steadily rising in developed countries. One out of 4–5 individuals now suffers from allergic diseases, and the improvement of environmental hygiene may be partly responsible for the increase in the prevalence of allergy [1]. In addition to the alarming increase of their prevalence, allergic diseases have also begun to be recognized as an aggravating factor for neuropsychiatric conditions such as anxiety, obsessive–compulsive disorders (OCD), and attention deficiency hyperactivity disorders (ADHD). In children diagnosed with AR, the prevalence of ADHD was higher (*p* < 0.001) in a large population study (*N* = 226,550) [2]. In another population study, ADHD individuals (*N* = 4692) revealed an increased risk of AR compared to non-ADHD controls (*N* = 18,768) [3].

Given the high prevalence of allergic disease in the general population, individuals with limited expressive language (LEL) are also expected to suffer from allergic diseases at a similar rate to the one observed in general population. Allergy symptoms are expected to elicit aberrant behaviors partly through pain and discomfort in these individuals. For example, nasal congestion and sinus headache may lead to self-injurious behaviors (SIBs) such as head banging, hand-biting, and nose-picking in individuals with LEL. Neuropsychiatric conditions such as anxiety and OCD can also be aggravated by allergic symptoms as described in the general population, which may further aggravate SIBs.

In general, treatment measures for allergic disease are safe and readily available. Thus, timely diagnosis and treatment for allergic diseases can be important for controlling behavioral and neuropsychiatric symptoms in individuals with LEL. Although diagnosis of allergic diseases is usually straight forward in typically developing children, patients with LEL may be far more difficult to diagnose and manage, due to the lack of effective speech and often aberrant behaviors. This may be especially true for children diagnosed with autism spectrum behaviors (ASD) who often exhibit challenging behaviors in clinical settings. It is our experience that in ASD children, worsening behavioral symptoms (typically hyperactivity and irritability) due to “flare-up” of allergic rhinitis during allergy seasons are frequently attributed inappropriately to the autism itself. With current medical education, primary care physicians and specialists are not well equipped for caring for patients with LEL. As a result, patients with LEL can be at high risk of being over-treated with neuropsychiatric medications for their behavioral symptoms, without consideration of the effects of allergy symptoms on their neuropsychiatric symptoms. This may be even more problematic in LEL subjects with severe allergy not responding to the first line allergy medications.

Currently in the United States, the second line allergy treatment is subcutaneous allergen immunotherapy (IT). This treatment requires subcutaneous injections of purified allergens in a doctor’s office weekly for the first 4–6 months, until reaching the maintenance doses of allergens, and then every 4 weeks thereafter. It may be difficult for LEL children with problematic behaviors to tolerate this time-consuming procedure. In addition, IT is not universally effective; individuals reactive to multiple allergens (so called poly-reactors) are less responsive to IT than those who react to limited numbers of allergens [4].

Another second line treatment option became available in the last decade in the form of humanized anti-IgE antibody (omalizmab) [5]. This murine monoclonal antibody inhibits binding of IgE to the IgE receptor expressed on effector cells (i.e., mast cells, basophils, eosinophils, and dentritic cells) and blocks allergen induced immune responses [5]. Its action is not allergen specific, and treatment is required every 2 or 4 weeks, depending on a patient’s total IgE level prior to treatment. Clinical benefits of omalizmab are manifested in a few weeks as opposed to IT, which takes several months to produce symptomatic relief. Thus omalizmab is likely to be better tolerated in LEL individuals with problematic behaviors. However, there is a small risk of systemic reaction or anaphylaxis with both aeroallergen IT and omalizmab (0.1–0.2 %) [4, 5].

We present 2 cases of such individuals (one with ASD and the other with developmental delay, congenital deafness, and other multiple congenital anomalies). In these 2 subjects, omalizmab treatment resulted in marked improvement not only in their naso-ocular symptoms, but also in the neuropsychiatric symptoms (especially difficult behaviors and cognitive activity), illustrating the profound effects of respiratory allergy in neuropsychiatric symptoms in subjects with LEL.

## Case presentation

### Case #1

Eleven-year-old Caucasian male who was previously evaluated in the pediatric allergy/immunology (A/I) clinic at 6 years of age for non-IgE mediated food allergy. He was one of triplets born following in vitro fertilization, and suffered from feeding difficulties, failure to thrive (FTT), and persistent gastrointestinal (GI) symptoms. ASD was diagnosed at 20 months of age by a developmental pediatrician at a nationally known autism diagnostic center; behavioral intervention measures were implemented before 2 years of age. However, he responded poorly to the behavioral intervention measures. At that time, he was diagnosed with food protein induced enterocolitis syndrome (FPIES) and advised to avoid offending food (mainly soy and cow’s milk protein). His FTT and GI symptoms resolved gradually following dietary interventions (soy-free, dairy-free diet). He also started to reveal slow but steady progress in his cognitive development.

However, after 8 years of age, his parents recognized marked exacerbation of his behavioral symptoms (anxiety, self-scripting, SIBs, and OCD behaviors) in spring months when his aeroallergen symptoms flared up. His spring allergy flare-up resulted in chronic sinusitis every year. The first line allergy medications (steroid nasal inhalers, a leukotriene receptor antagonist, and topical ophthalmic solutions for ocular allergy) failed to control his naso-ocular symptoms. At 11 years of age, he presented in our clinic with marked exacerbation of behavioral symptoms starting in spring. His parents also reported that school teachers diagnosed him having developmental regression with loss of once-acquired cognitive skills. At the time of secondary presentation, his cognitive skills were less than 1 percentile by an evaluation conducted at his school; his teachers thought that he was unable to comprehend a simple arithmetic question such as 1 + 1.

His parents were concerned with possible pediatric acute-onset neuropsychiatric syndrome, secondary to his worsening OCD/anxiety, frequent temper tantrums, and SIBs (banging heads, biting hands). In fact, he was expelled from his school due to his problematic behaviors. Prior to presenting in our clinic for the second time, he had been evaluated by several neurologists for possible metabolic, genetic, and infectious diseases that could have been associated with drastic changes in his behavioral symptoms. Neurological workup including imaging studies and EEG was unrevealing, ruling out organic, metabolic, genetic, or infectious etiology. The only notable clinical findings were seasonal allergy symptoms worsening in spring with complications of sinusitis every year. His aeroallergen reactivity was confirmed, with presence of allergen-specific IgE against tree pollens (maple, birch, oak, elm, walnut, cottonwood, and white ash) in the serum. He was also moderately reactive to grass and weed pollens. Because of his lack of responses to the first line allergy medications, omalizmab (300 mg every 4 weeks) was started at 12 years of age, one month prior to the start of spring allergy season, since omalizmab is approved for children ≥12 years old in the United States. Allergen IT was deferred because of his difficult behaviors and his reactivity to multiple pollen allergens, which makes it less likely that he would respond favorably to allergen IT.

**Fig. 1:**
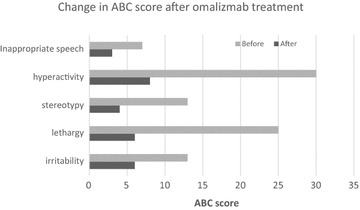
Aberrant Behavior Checklist (ABC) subscale scores before and after the omalizmab treatment in the case #1

Omalizmab effectively controlled his naso-ocular symptoms within 1–2 months. After starting omalizmab, behavioral symptoms were much less variable in spring/summer months (Fig. 1). His parents also began to suspect that he was more capable than the level at which he was initially evaluated at his school. Eventually, he became able to attend another school where he made rapid progress academically. He was found to be capable of 3-digit addition/subtraction, multiplication, fraction, and division 18 months after starting omalizmab treatment. Omalizmab dose was then increased due to suboptimal control of allergy symptoms, which was likely associated with the onset of puberty and a subsequent growth spurt. His AR symptoms became better controlled with an increased dose of omalizmab (300 mg every 2 weeks), which resulted in further improvement of his behavioral symptoms, resolution of SIB, and excellent academic achievement. He was also found to be talented in music with perfect pitch. Improvement of his behavioral symptoms are noted in changes in ABC scores as shown below. Unfortunately, interruption of omalizmab treatment due to delay in insurance approval, following changes in his insurance coverage, resulted in worsening of his behavioral symptoms in 2015 at 16 years of age. This was again resolved after resuming omalizmab treatment.

### Case #2

10-year-old male with mixed ethnicity (half Asian/half Caucasian) was previously evaluated in the Pediatric Allergy/Immunology Clinic for delayed type food allergy around 4–5 years of age. He was born with multiple congenital anomalies including bilateral neurosensory hearing loss, agenesis of right kidney, and congenital heart disease (CHD) (pulmonary atresia, ventricular septal defect, and patent ductus arteriosus). He had successful surgical repair of his CHD in his infancy. He has global developmental delay and has continued to be non-verbal. His GI symptoms were resolved with dietary intervention, but he continued to be followed up in our clinic secondary to asthma and seasonal allergy symptoms. His major allergens were tree pollens, with significantly elevated allergen-specific IgE against oak and birch pollens (Class V and class VI). He was also reactive to multiple tree, weed, and grass pollens. His allergy symptoms were initially reasonably well controlled with the first line allergy medications including steroid nasal inhaler, olopatadine ophthalmic solution, and montelukast, a leukotriene receptor antagonist. Anti-histamines were also used as needed. However, after 10 years of age, his spring allergy symptoms became more significant, with new onset of facial tics/anxiety/OCD behaviors, worsening hyperactivity and less focusing, resulting in disturbance of his school performance. Since he had pending surgical correction of his CHD at 13–14 year of age, along with his reactivity to multiple pollens, and his disability, he was considered to be at high risk of allergen IT. We thus elected to place him on omalizmab treatment at 12 years of age, since omalizmab is approved for children ≥12 years old in the United States. Despite his aberrant behaviors, he tolerated omalizmab remarkably well with almost complete resolution of AR symptoms within 1–2 months after starting omalizmab. In parallel to resolution of AR symptoms, facial tics, anxiety, and OCD behaviors resolved, and his behavioral symptoms returned to his baseline. He then started to adapt sign language, gaining better communication skills.

## Conclusions

This study presents 2 LEL cases of individuals suffering from severe aeroallergen allergy which required the second line allergy treatment, omalizmab. Remarkable findings in these 2 LEL cases are marked attenuation of their problematic neuropsychiatric symptoms and subsequent improvement in the cognitive development, once respiratory allergy symptoms were under control. These 2 LEL cases revealed that respiratory allergy can manifest profound detrimental effects on neuropsychiatric symptoms and cognitive activities in LEL individuals, illustrating the importance of optimal control of respiratory allergy symptoms in LEL subjects.

As detailed in the background section, the aggravating effects of respiratory allergy on neuropsychiatric symptoms has been convincingly shown by multiple epidemiological studies in the general population. This finding is also very likely to be applicable for individuals with LEL. Unfortunately, IgE-mediated allergic diseases tend to be under-diagnosed and/or under-treated in LEL subjects, based on our clinical experience. This is partly due to their impaired communication skills, but also to the difficulty of recognizing an association between behavioral symptoms and allergy exacerbation. The 2 cases presented illustrate a need for high index of suspicion of underlying treatable medical conditions when assessing marked changes in neuropsychiatric symptoms in individuals with LEL.

It is relatively easy to try the first line allergy medications for symptomatic relief for suspected allergy in LEL subjects with or without supporting laboratory results. However, if their responses are suboptimal, a question will arise whether it is necessary to proceed to the second line allergy treatment because of the cost and time required for such procedures. In addition, the presence of problematic behaviors may raise doubt concerning how the subject will tolerate the proposed procedures. This was in fact our initial concern. However, supporting laboratory data and well documented seasonal changes in problematic behaviors, made us proceed to the second line allergy treatment. The presented 2 cases revealed remarkably favorable responses, and illustrate the importance of optimal control of allergy symptoms in LEL patients in terms of behavioral symptoms, and even cognitive development. Clinicians should seek the second line allergy treatment measures if there is sufficient clinical evidence that supports expected efficacy of such measures.

## Consent

Parents of these 2 presented cases have given their consent for the case reports to be published. The signed consent form was obtained prior to the submission of this manuscript.

## Abbreviations

ADHD: Attention deficit hyperactivity disorder
ASD: Autism spectrum disorder
CHD: Congenital heart disease
EEG: Electroencephalography
FPIES: Food protein induced enterocolitis syndrome
FTT: Failure to thrive
GI: Gastrointestinal
IgE: Immunoglobulin E
IT: Immunotherapy
LEL: Limited expressive language
OCD: Obsessive compulsive disorder
SIBs: Self-injurious behaviors
